# Ocular and neural genes jointly regulate the visuospatial working memory in ADHD children

**DOI:** 10.1186/s12993-023-00216-9

**Published:** 2023-09-01

**Authors:** Yilu Zhao, Yuanxin Zhong, Wei Chen, Suhua Chang, Qingjiu Cao, Yufeng Wang, Li Yang

**Affiliations:** 1 Peking University Sixth Hospital, Peking University Institute of Mental Health, National Clinical Research Center for Mental Disorders, Peking University Sixth Hospital), NHC Key Laboratory of Mental Health (Peking University), 51 Huayuan Bei Road, Beijing, 100191 China; 2 Department of Psychiatry, Li Ka Shing Faculty of Medicine, The University of Hong Kong, Hong Kong, SAR China; 3 Sichuan Provincial Center for Mental Health, The Center of Psychosomatic Medicine of Sichuan Provincial People’s Hospital, University of Electronic Science and Technology of China, Chengdu, Sichuan, China

**Keywords:** Attention deficit hyperactivity disorder, Visual-spatial working memory, Genome-wide association study, Polygenic risk score, Visual system

## Abstract

**Objective:**

Working memory (WM) deficits have frequently been linked to attention deficit hyperactivity disorder (ADHD). Despite previous studies suggested its high heritability, its genetic basis, especially in ADHD, remains unclear. The current study aimed to comprehensively explore the genetic basis of visual-spatial working memory (VSWM) in ADHD using wide-ranging genetic analyses.

**Methods:**

The current study recruited a cohort consisted of 802 ADHD individuals, all met DSM-IV ADHD diagnostic criteria. VSWM was assessed by Rey-Osterrieth complex figure test (RCFT), which is a widely used psychological test include four memory indexes: detail delayed (DD), structure delayed (SD), structure immediate (SI), detail immediate (DI). Genetic analyses were conducted at the single nucleotide polymorphism (SNP), gene, pathway, polygenic and protein network levels. Polygenic Risk Scores (PRS) were based on summary statistics of various psychiatric disorders, including ADHD, autism spectrum disorder (ASD), major depressive disorder (MDD), schizophrenia (SCZ), obsessive compulsive disorders (OCD), and substance use disorder (SUD).

**Results:**

Analyses at the single-marker level did not yield significant results (5E−08). However, the potential signals with P values less than E−05 and their mapped genes suggested the regulation of VSWM involved both ocular and neural system related genes, moreover, ADHD-related genes were also involved. The gene-based analysis found *RAB11FIP1*, whose encoded protein modulates several neurodevelopment processes and visual system, as significantly associated with DD scores (P = 1.96E−06, P_adj_ = 0.036). Candidate pathway enrichment analyses (N = 53) found that forebrain neuron fate commitment significantly enriched in DD (P = 4.78E−04, Padj = 0.025), and dopamine transport enriched in SD (P = 5.90E-04, Padj = 0.031). We also observed a significant negative relationship between DD scores and ADHD PRS scores (P = 0.0025, Empirical P = 0.048).

**Conclusions:**

Our results emphasized the joint contribution of ocular and neural genes in regulating VSWM. The study reveals a shared genetic basis between ADHD and VSWM, with GWAS indicating the involvement of ADHD-related genes in VSWM. Additionally, the PRS analysis identifies a significant relationship between ADHD-PRS and DD scores. Overall, our findings shed light on the genetic basis of VSWM deficits in ADHD, and may have important implications for future research and clinical practice.

## Introduction

Attention deficit hyperactivity disorder (ADHD) is a highly heritable disorder in children, with an approximately 7.2% prevalence [1]. Except for the main clinical features (i.e., age-inappropriate attention deficit, hyperactivity, and impulsivity), ADHD cases often display multiple neuropsychological deficits, including working memory (WM), planning, vigilance and inhibition [2].

Working memory is the capacity to manipulate some information and maintain other information simultaneously, including auditory-verbal working memory and nonverbal working memory [3]. Visual-spatial working memory (VSWM) is a kind of nonverbal working memory, refers to the ability of temporarily holding and manipulating visual and spatial information in the mind for use in ongoing tasks. Meta-analyses indicated that VSWM was impaired in ADHD individuals [4–7], and previous evidence showed a positive relationship between the severity of ADHD symptoms and VSWM deficits [8–10]**,** suggesting VSWM might serve as one promising endophenotype for ADHD [4].

The Rey-Osterrieth Complex Figure (RCFT) is a validated and widely used measure of VSWM, developed by Rey [11]. The RCFT is capable of distinguishing between various aspects of VSWM, including the ability to retain the visual details of the figure, the ability to organize and integrate the different parts of the figure, and the ability to manipulate the figure mentally [12, 13]. Previous studies have consistently shown that ADHD individuals exhibit compromised performance on the RCFT task compared to typically developing (TD) individuals [14–16]. Specifically, deficits have been observed in the delayed scores and the organizational scores, indicating impairments in short-term working memory, global visual processing, and working memory integration [10, 14].

Visual-spatial working memory (VSWM) has been shown to be moderately to highly heritable by twin studies [17, 18]. At the molecular level, although the specific gene basis is still unknown, evidence from candidate gene-based analyses suggested that the dopaminergic circuit-related genes, which served as a main etiological pathway in ADHD, were involved [19]. Previous studies suggested the dopamine transporter (DAT) is a promising candidate risk gene in the etiological process of ADHD and has also been found to affect WM [20–23]. Dopamine receptor D1 (*DRD1*) gene, in which SNPs rs4532 and rs265978 moderated working memory improvements over development, also predicted ADHD symptoms reduction [24]. The Associations between superior VSWM function and carrying Met in the Valine158Methionine (Val158Met) SNP (i.e., the rs4680) were also demonstrated [25–27].

However, recent evidence suggests that causal variants involved in complex traits are not restricted to specific core pathways [28]. Consistent with this notion, recent GWAS studies in VSWM also revealed a more complicated landscape, with not only dopaminergic genes, but also genes associated with synaptic plasticity, apolipoprotein, neurodegenerative disorders, and others being involved [29–32]. A GWAS study by Blokland et al. failed to test significant SNP associated with WM-related brain regions, however, nominal signals were found within genes BANK1 and FOXQ1, which were involved in dopaminergic circuit and DNA binding, respectively [31]. Other two GWAS study found genes potentially associated with WM or visual-spatial ability at the 10^–6^ significant level, with implications in several psychiatric disorders and adrenergic receptor signaling pathway [29, 30]. A study published in 2022 found significant SNPs associated with verbal short-term memory, involving gene APOE and CDH18, which were responsible for Alzheimer’s disorders and synaptic plasticity, respectively [32].

The manipulation of VSWM involves coordinated interactions of a distributed neural network, with the frontal lobe playing a central role as a control structure [33–36] Dopamine transmission in the frontal cortex is critically for VSWM [37, 38], and the frontal lobe might serve as an important mediate factor between gene and WM deficits in ADHD [39]. An inverted U-shaped curve has been demonstrated in the relationship of frontal lobe functioning and WM performance, whereby both deficient and excessive amounts of prefrontal dopamine activity predict poor task performance [40].

The prefrontal lobe and dopaminergic circuit also involved in WM treatment response. Prior evidence has shown that improvements of VSWM after behavioral training was associated with changes in the density of cortical dopamine D1 receptors in prefrontal lobe [41]. Methylphenidate, a most widely used pharmaceutical treatment for ADHD, acts to increase the synaptic concentration of dopamine and noradrenaline could improve WM by altering regional cerebral flow (rCBF) in frontal and parietal lobes [42].

In conclusion, VSWM deficits in ADHD have a complex genetic basis that has not been fully understood. Previous candidate-gene based studies have identified that ADHD related genes involved in the dopaminergic circuit in frontal lobe are associated with VSWM deficits in TD and ADHD. As a primary neuropsychological function and endophenotype, VSWM is considered to be less genetically complex than clinical phenotypes. Thus, deconstructing the genetic basis underlying VSWM might shed light on illuminating the pathophysiological processes of ADHD, helps establish objective neurocognitive diagnostic modalities and holds promises for the development of precise behavioral therapies.

The current study had two main objectives: (1) to identify genes associated with VSWM in individuals with ADHD using a genome-wide approach and explore the relevant biological processes, especially in dopaminergic circuit and forebrain development; (2) to disentangle overlapping and distinct genetic structures underlying different visuospatial processes involved in different parts of the RCFT task.

## Materials and method

### Participants

The cohort, which consisted of 802 ADHD cases (684 males, 85.3%) aged between 6 and 16 (9.9 ± 2.4) was recruited from the Child and Adolescent mental health center at Peking University Sixth Hospital. All patients met the diagnostic criteria of the Statistical Manual of Mental Disorders-IV (DSM-IV) for ADHD. The diagnosis was first made by a child psychiatrist and then confirmed by semi-structural interview using the Chinese version of KSADS-PL [43]. Patients with epilepsy, schizophrenia, pervasive development disorder or mental retardation (IQ < 70) were excluded [44]. Chinese Children version of the Wechsler Intelligence Scale (C-WISC, third edition) was used to assess IQ [45, 46]. The protocol was reviewed and approved by the Ethics Committee of Peking University Health Science Center. Written informed consent was obtained from the parents and from both the participants themselves and their parents if they were over 8 years old.

### Neuropsychological test: rey-osterrieth complex figure test (RCFT)

All subjects finished the RCFT to evaluate VSWM. Subjects were instructed to remember the RCFT figure within 30 s and subsequently draw what they remembered immediately (without intervening distraction) and then a 20–30 min' delay (other tests were conducted during this delay) [10]. This test allowed us to observe subjects’ immediate and delayed visual-spatial working memory performances [14]. Detail and structure scores were rated using 36-point and 6-point, respectively. The 36-point scoring system was initially devised by Osterrieth [50] and adapted by Booth [75], which could reflect both local and global VSWM. In the 36-point system, each figure is scored using 18 features: two points are given if the feature is placed correctly, and one point given if it is incomplete or placed poorly. The 6-point scoring system was devised by Binder [51], evaluated the accuracy and completeness for constructing five configural elements and the addition of the base rectangle, which was used to evaluate the organizational strategies during RCFT task and global processing.

### Genotyping quality control

The Omega DNA extraction Kit (Omega Bio-tek Inc., Doraville, GA) was used to extract genomic DNA from peripheral blood. The genomic DNA was genotyped using the Affymetrix 6.0 array with standard protocol at Capital Bio Ltd (Beijing) [44]. For quality control at the individual level, we excluded those with per-individual autosomal heterozygosity > 5 SD away from the mean, missing age or IQ information, as well as with a per-individual call rate < 95%. For quality control at the SNP level, we included SNPs with call rate > 98%, Hardy–Weinberg equilibrium test P > 0.001 [47], and minor allele frequency (MAF) > 5%. After quality control, 802 ADHD patients remained. We then used MACH-admix 1.0 [48] to impute non-genotyped SNPs, using the ASN data from the Integrated Phase 1 Release of the 1000 Genome Projects (GRCh37/hg19) as the reference panel. Imputed SNPs with a squared correlation between imputed and true genotypes r^2^ < 0.6 or SNPs with minor allele frequency < 0.05 were removed.

Principle component analysis (PCA) was performed to check population stratification. It was conducted using the SNPs with low linkage disequilibrium (LD, MAF > 0.05 and r^2^ < 0.05 for each pair of SNPs) that were outside the 5 long-range LD regions using the EIGENSOFT 4.2 software [49], and validated by the Tracy-Widom test. There was no significant population stratification. Only the top 1 eigenvector was used as a covariate in subsequent statistical analyses.

### Genome wide association analyses (GWAS) and annotation

The association between SNPs and the RCFT scores was conducted using the additive linear regression model by PLINK version 1.9 with age, sex and eigenvector one from principle component analysis as covariates [50–52] In order to thoroughly address potential confounding effects of age, we conducted additional tests to examine the potential interaction relationships between age and genotypes (GWAS P-value less than 1E−05).

We used three methods for gene mapping: positional mapping, eQTL mapping, and chromatin interaction mapping. For positional mapping, we used a maximum distance of 10 kb [53]. The eQTL data and additional annotation data based on Hi-C results were obtained from 3D SNP and GTEx database ([54], https://gtexportal.org/home/). The Genecards website was used to check the function of genes [55, 56].

### Gene-based analysis

We used Multi-marker Analysis of GenoMic Annotation (MAGMA) [57] for gene-based analysis. As recommended by de Leeuw et al. [57], we utilized the genotypic data as inputs instead of using GWAS summary statistics. The analytic procedure is as follows: SNPs were mapped to corresponding genes based on the NCBI 37.3 gene definitions, extending gene footprints by an additional 35 kilobase (kb) up- and 10 kb downstream as default. After SNP annotation, there were 18,177 genes that were covered by at least one SNP. Bonferroni correction was used, setting the two-side genome-wide threshold for significance at 2.75E−06.

### Candidate pathway-based analysis

Since previous evidence emphasized the critical role of dopaminergic circuits and the frontal lobe in the regulation of VSWM; and the current GWAS results indicated participation of ocular system (described in the result section). The current study included 53 Gene ontology biological process (GOBP) pathways involved in frontal lobe/forebrain (n = 14), dopaminergic circuit (n = 15), and eye/visual perception (n = 24) as candidate pathways. Using MAGMA, we tested the association of candidate pathways with four RCFT scores. We adopted a Bonferroni correction, setting the threshold of significance P < 9.43E−04.

### Protein–protein interaction (PPI) network construction

GeneMANIA (http://www.genemania.org) was adopted to construct the network of RCFT interactive proteins based on physical interactions, co-expression, predicted, co-localization, pathway, genetic interactions, and shared protein domains [58]. Visualization was implemented using Cytoscape [59].

### Polygenic risk score (PRS)

Using the current cohort as target, we calculated PRSs using latest Psychiatric Genomic Consortium (PGC) GWAS statistics of six psychiatric disorders as base data, including ADHD, autism spectrum disorder (ASD), obsessive compulsive disorder (OCD), major depression disorder (MDD), schizophrenia (SCZ), bipolar disorder (BIP), and substance use disorder (SUD) [60–66]. PRSice-2 [67] were used to calculate PRS scores and perform the high-resolution scoring method to select the most precise threshold for SNPs associated with each RCFT score (with lower and upper threshold as 0 and 0.5 respectively and the interval as 0.0001). The significance of the regression results was corrected by a permutation test with 10,000 replicates, an empirical P value less than 0.05 will be taken as significant. Pearson correlation pho was calculated between each index and psychiatric PRS scores and shown in a heatmap to aid in visualization.

## Results

A total of 802 ADHD cases were included in the study, comprising 684 boys (85.3%) with an average age of 9.9 ± 2.4 years, and an average IQ was 104.5 ± 15.7. The average points for DD (9.1 ± 0.2), DI (9.5 ± 0.2), SD (2.7 ± 0.1), and SI (2.9 ± 0.1) were also measured (Table 1).

**Table 1:** Demographic characteristic and VSWM profile of the current cohort

Num of participants	Age (years)	Sex (boy: girl)	IQ	REYDD	REYDI	REYSD	REYSI
802	9.9 (2.4)	684:118	104.5 (15.7)	9.1 (0.2)	9.5 (0.2)	2.7 (0.1)	2.9 (0.1)

**Fig. 1:**
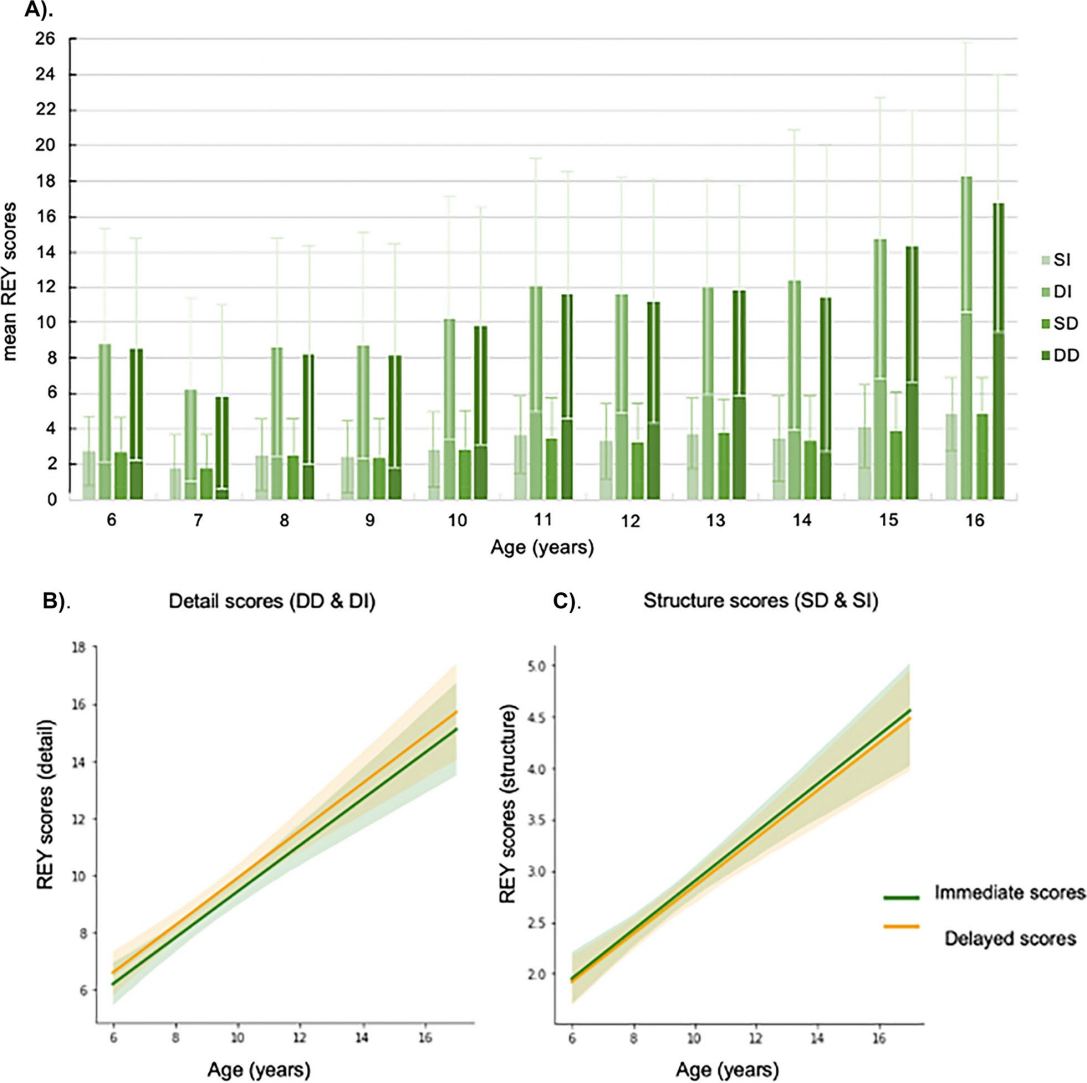
**A** Bar plots showing RCFT scores in different age groups (the residual bars denote standard error of mean RCFT scores); **B**, **C** Plots presenting the positive relationships between RCFT scores and ages (the shaded area represents the 95% confidence interval of the regression)

Furthermore, we found significant positive relationships between ages and all four RCFT scores (P_bon_ < 0.05), suggesting that VSWM functions undergo dynamic changes over time (Fig. 1).

### SNP, gene level associations and protein networks

Genome-wide association analyses, after quality control, were conducted for four RCFT scores. The SNP level association results did not reach genome-wide significance (P < 5e−8). However, we identified 26 potential risk loci with P < 1e−05 of DD, DI, SD, and SI (Table 2). None of the 26 risk loci showed potential interaction relationships between age and genotypes (interaction P values in all variants were larger than 10E−03).

**Table 2:** Nominal significant loci associated with four RCFT scores

SNP	CHR	BP	A1	Beta	P-value	Mapped genes (location)	Mapped genes (eQTL and HiC)
REYDD							
rs56111147	14	96,153,765	C	−2.368	1.34E−06	TCL1B	TCL1A, TCL6 (HiC), TCL1B (eQTL)
rs277785	8	94,335,268	A	1.36	4.12E−06	LOC107986956	C8orf87 (Hi-C)
rs141358499	6	28,803,174	T	2.914	6.25E−06	NA	ZSCAN31, ZNF603P, ZSCAN8 (eQTL & HiC), AL022393.9, ZSCAN16 (HiC)
REYDI							
rs56111147	14	96,153,765	C	−2.656	1.25E−07	TCL1B	TCL1A, TCL6 (HiC), TCL1B (eQTL)
rs150898617	6	28,834,324	A	2.769	9.97E−07	RPL13P	ZSCAN31, ZNF192P1, ZNF602P, ZSCAN16 (eQTL); RPSAP2, ZNF311, RN7SL471P (HiC)
rs637487	1	191,459,085	A	−1.58	1.03E−06	NA	NA
rs857125	1	57,205,340	C	1.843	1.70E−06	C1orf168	C1orf168, PRKAA2 (Hi-C)
rs7667462	4	153,841,204	A	1.293	4.68E−06	FHDC1	ARFIP1 (eQTL)
rs4394025	4	76,497,433	C	−2.193	5.73E−06	NA	G3BP2, CDKL2 (eQTL & HiC); C4orf26, PARM1, RCHY1, THAP6, SO1 (HiC)
REYSD							
rs7594919	2	207,999,571	C	−0.648	3.29E−06	KLF7	KLF7, MIR2355 (HiC)
rs12981571	19	8,175,595	C	0.133	3.50E−06	FBN3	FBN3, CCL25, CERS4, ELAVL1 (HiC)
rs2778915	9	101,385,524	C	0.484	7.41E−06	GABBR2	GABBR2 (HiC)
rs12686018	9	18,879,776	A	−0.565	7.59E−06	ADAMTSL1	NA
rs72909972	2	157,109,471	C	0.505	9.65E−06	LINC01876	NR4A2
REYSI							
rs4932303	15	90,752,952	C	1.030	1.83E−06	SEMA4B	CIB1, SEMA4B (HiC & eQTL), GABARAPL3, GDPGP1, IDH2, NGRN (HiC), ZNF774 (eQTL)
rs12629965	3	128,422,658	A	−0.461	3.99E−06	NA	RPN1, POU5F1P6 (eQTL)
rs62188624	2	208,017,033	A	−0.651	4.18E−06	KLF7	KLF7, CREB1 (HiC)
rs56111147	14	96,153,765	C	−0.785	5.89E−06	TCL1B	TCL1A, TCL6 (HiC), TCL1B (eQTL)
rs2826536	21	22,187,947	C	0.415	6.29E−06	NA	LINC00320, NCAM2 (HiC)
rs4472720	1	187,613,231	G	−0.760	7.01E−06	NA	NA
rs12981571	19	8,175,595	C	0.593	7.40E−06	FBN3	FBN3, CCL25, CERS4, ELAVL1 (HiC)
rs17178718	17	5,551,690	C	0.812	7.64E−06	NA	NLRP1 (eQTL)
rs2761312	10	95,052,262	C	−0.731	7.83E−06	NA	MYOF (sQTL)
rs2088308	3	82,839,698	A	−0.432	8.1E−06	NA	NA
rs2705168	12	48,959,389	G	0.512	9.29E−06	LALBA	LALBA (HiC)
rs7071815	10	133,871,035	G	0.479	9.65E−06	JAKMIP3	JAKMIP3 (HiC & eQTL)

**Fig. 2:**
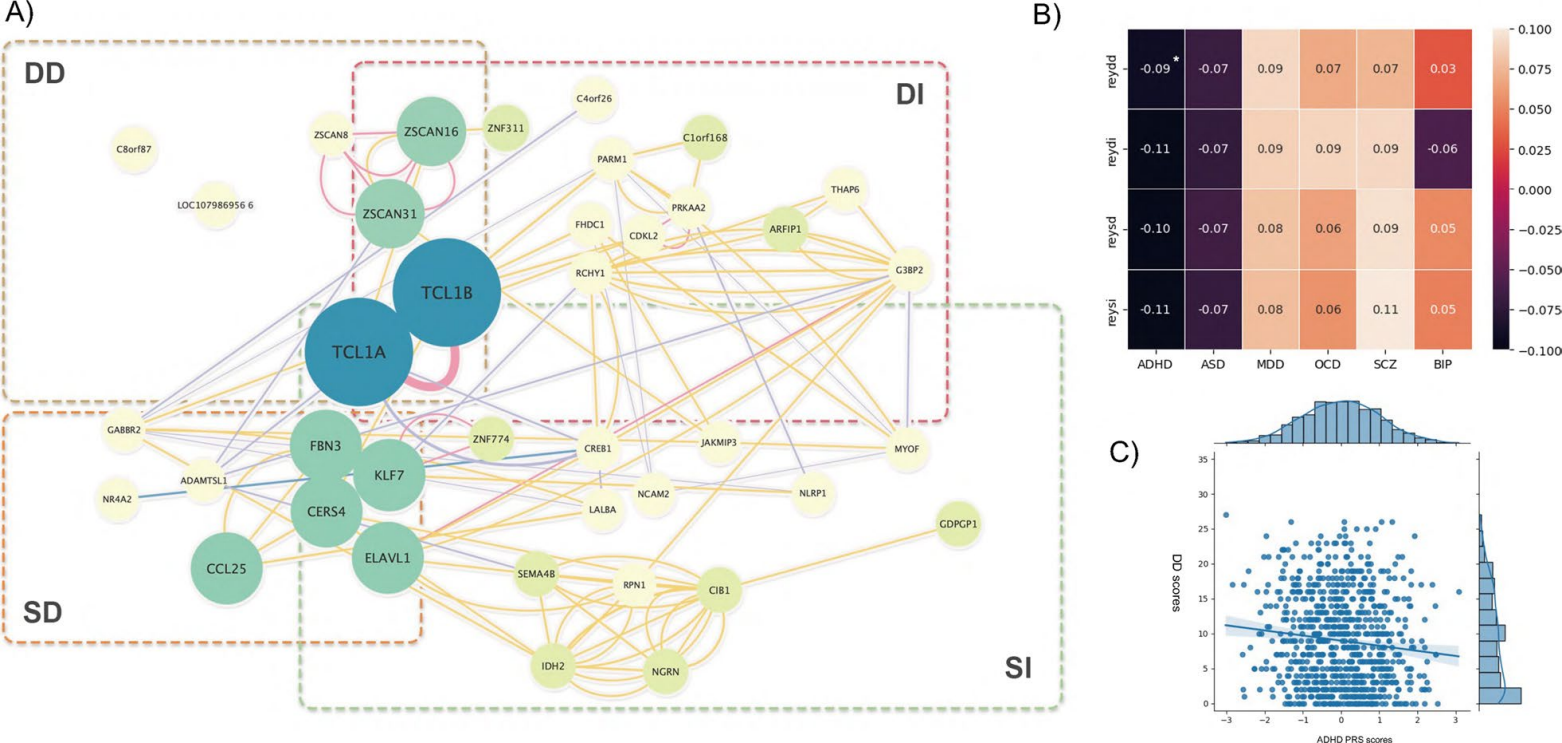
Results of network and polygenic risk score analyses. **A** Gene interaction networks. Nodes and edges were constructed using relevant genes and gene interactions detected by GeneMANIA. Genes involved in four indexes exhibited a cooperative pattern, and TCL1A and TCL1B were identified as overlap nodes for three indexes (DD, DI, SI). Node size was determined by −log P (single–marker based P value), with sizes doubling if SNPs were involved in more than one index. Edges were constructed based on correlations between genes, with line width indicating the strength of correlation; **B** correlation heatmap between four RCFT scores and PRS scores for five psychiatric disorders. White asterisk denotes significant correlations (empirical P value less than 0.05); **C** Scatter plots for ADHD PRS scores and REY-DD, significant negative relationships were revealed

We identified 3, 6, 5, and 12 SNPs potentially associated with DD, DI, SD, and SI, respectively. Candidate risk genes for each locus were mapped and prioritized accordingly (Table 2). Interestingly, locus rs56111147 was potentially associated with three indexes except for SD, and it was located in the gene *TCL1B*, which has been previously linked to myopia [68]. Inspection of the mapped genes revealed that relevant genes were mainly related to the ocular and neural system (Fig. 2A). Additionally, delayed scores were associated mostly with neural system related genes, while most of the oculus related genes were relevant to immediate scores.

Gene-based analysis identified a gene Rab11-family interacting protein one (*RAB11FIP1*) significantly associated with DD score (P = 1.96E-06, P_adj_ = 0.036).

Furthermore, in order to detect the genetic interaction networks between these four indexes, we constructed the protein networks connected by the mapped candidate genes (Fig. 2A). Both distinctive and common parts in the network were highlighted. Gene *TCL1B* and *TCL1A* were the central hub protein shared by DD, DI, and SI, and interacts with genes involved in DI, SD, and SI (*FHDC1, ADAMTSL1, PARM1, CERS4, CDKL2, ZSCAN16, CREB1*). Genes of zinc protein family were also highlighted as top hub shared by DD and DI (*ZSCAN16, ZSCAN31*).

### Candidate pathway analyses

We identified two pathways significantly involved in the RCFT. The forebrain neuron fate commitment pathway was enriched in DD (P = 4.78E−04, P_adj_ = 0.025), and the dopamine transport pathway was enriched in SD (P = 5.90E−04, P_adj_ = 0.031).

Although no pathways associated with DI and SI scores passed the multiple test correction, forebrain neuron fate commitment and dopamine transport were implied to be potentially relevant to DI (P = 1.16E−03, 2.55E−03); dopamine transport, adenylate cyclase inhibiting dopamine receptor signaling pathway and forebrain neuron differentiation potentially enriched in SI (P = 2.16E−03, 4.73E−03, 7.62E−03) (Table 3).

### Polygenic risk score

A significant relationship was observed between DD and ADHD PRS scores, with an uncorrected P value of 0.0025, and an empirical P value of 0.035 (Fig. 2B). Although potential relationships between other RCFT scores and PRS were suggested, however, failed to pass the permutation test. The correlation heatmap was shown in the Fig. 2C.

## Discussion

The present study employed wide-range genetic analyses to explore the genetic basis underlying VSWM in ADHD. Although no SNP reached the genome wide significance, following the previous evidence demonstrated that missing heritability could be explained by the SNPs far below the genome-wide significance [28], we investigated the nominal significant SNPs (P < 1E−05) and highlighted a joint modulation pattern implemented by ocular and neural genes. And a differential pattern under four indexes might exist, with oculus related genes were implied to be more involved and neural system related genes less involved in detail scores compared with immediate scores. Gene-based analysis identified a significant brain-expressed gene *RAB11FIP1* (UniProt & lifeMap Discovery), which is critical for several neurodevelopmental process and visual pathways, associated with DD score. Dopaminergic circuit and forebrain development were suggested to be involved in SD and DD, respectively. Shared genetic basis between ADHD and VSWM were indicated, as the current study found the involvement of ADHD-related genes underlying VSWM and significant relationship between ADHD PRS and DD scores.

1. Ocular and neural system related genes jointly modulate VSWM.

Working memory is a complex and intricate system [69]. Neurobiological studies found that VSWM mainly includes two stages: (1) Visual perception, during which a set of physical signals enter the visual system and the brain interprets the electromagnetic signals and produces “a percept” or interpretation of the physical reality. In this process, neurons in the visual pathway, posterior early sensory and association areas are involved; (2) mnemonic representation, i.e., the brain, mainly PFC produces and maintains a mental representation of a percept when unavailable to the senses [70–72] The above findings from neurobiological studies suggested that the visual organ, the visual pathway, and the brain (mainly frontal lobe) are the basic biological structure underlying VSWM.

In line with previous knowledge, the current study provided further evidence that ocular and neural genes jointly modulate visual-spatial working memory through a comprehensive set of genetic analyses. Using GWAS method (P < 1e−05) (Table 2), we found that most relevant genes are brain-expressed (GTEx v8), and several genes are related to neurodevelopmental process and psychiatric disorders (Zinc finger family coding genes, *C8orf87, RPL13P, G3BP2, GABBR2, LINC01876, SEMA4B, RPN1* [73–85]); Meanwhile, multiple genes were found to be involved in the ocular system, with gene *NLRP1* enhancing in Muller glia cells, gene *NR4A2* and gene *IDH2* over-expressing in the eye (LifeMap), and genes *TCL1B, TCL1A, TCL6, KLF7, FBN3, C1orf168, ADAMTSL1, MYOF, SEMA4B, CIB1, CREB1, CERS4* and *NLRP1* being involved in eye diseases and eye measurements, such as myopia [68], [86–93]glaucoma [88, 94], retinopathy [95], pinguecula [96], uveitis [97], corneal resistance factor, exploratory eye movement [86], and intraocular pressure measurement [87, 98].

Moreover, our findings suggested that portions of ocular genes and neural genes underlying the four indexes might vary. Compared with delayed recall, genes underlying immediate recall might be slightly more related to the visual system and less related to the neural system (Fig. 2A). *KLF7, FBN3, SEMA4B, CIB1, NLRP1, NR4A2* and *MYOF,* which were suggested only in immediate recall, were associated with visual system. However, in delayed recall task, only *TCLs (TCL1A, TCL1B, TCL6)*, which also related to immediate recall (Table 3), and *C1orf168* were involved in visual function.

**Table 3:** Top 5 enriched gene ontology (GO) pathway in four indexes

GO pathway	N gene	Beta	SE	P
REYDD				
Forebrain neuron fate commitment	9	1.1369	0.3441	4.78E−04*
Dopamine transport	47	0.20113	0.13196	6.37E−02
Wnt signaling pathway involved in midbrain dopaminergic neuron differentiation	11	0.39499	0.29188	8.80E−02
Midbrain dopaminergic neuron differentiation	16	0.29743	0.24711	1.14E−01
Regulation of dopamine uptake involved in synaptic transmission	7	0.33785	0.29334	1.25E−01
REYDI				
Forebrain neuron fate commitment	9	1.0519	0.34518	1.16E−03
Dopamine transport	47	0.37079	0.13236	2.55E−03
Forebrain morphogenesis	11	0.63099	0.30332	1.88E−02
Synaptic transmission dopaminergic	28	0.28778	0.14888	2.66E−02
Adenylate cyclase inhibiting dopamine receptor signaling pathway	8	0.68815	0.35835	2.74E−02
REYSD				
Dopamine transport	47	0.43833	0.1351	5.90E−04*
Forebrain neuron fate commitment	9	0.97901	0.35241	2.74E−03
Dopamine secretion	36	0.37745	0.16311	1.03E−02
Forebrain morphogenesis	11	0.58913	0.30966	2.86E−02
Forebrain regionalization	21	0.37972	0.22967	4.91E−02
REYSI				
Dopamine transport	47	0.3859	0.13521	2.16E−03
Adenylate cyclase inhibiting dopamine receptor signaling pathway	8	0.95016	0.36607	4.73E−03
Regulation of dopamine uptake involved in synaptic transmission	7	0.73861	0.30058	7.00E−03
Forebrain neuron differentiation	46	0.37687	0.15529	7.62E−03
Forebrain neuron fate commitment	9	0.80482	0.3527	1.13E−02

*Statistical significance with P-value < 9.43E−04

The results of gene-based analysis further support the current findings from GWAS analysis. Specifically, we identified an association between DD scores and the *RAB11FIP1* gene, which was involved in both ocular and neural processes. *RAB11FIP1* is over-expressed in brain, encodes one of the Rab11-family interacting proteins (Rab11-FIPs), which plays a crucial role in the Rab-11 mediated recycling of vesicles. Proteins in the Rab11-FIPs family are known to modulate various neurodevelopmental processes, such as dendritic arborization, neurite pruning, synaptic plasticity, and spatial memory formation [99, 100]. Moreover, previous evidence indicated that these proteins are also involved in the visual system, as they have been implicated in retina neurogenesis [101, 102], photoreceptor cells development and maintenance [103, 104].

(2) Shared genetic basics between ADHD and VSWM.

The current studies identified genes shared by VSWM and ADHD, including *C8orf87* (DD) [105], *ZSCAN31* (DD, DI) [77, 83], *RPL13P* (DI) [85], *FBN3* (SD, SI) [74], and *LINC01876* (SD) [81, 83]. *C8orf87* was identified in a meta-analysis of ADHD GWAS studies [105]. *ZSCAN31*, a member of Zinc finger protein family, encodes a protein containing multiple C2H2-type zinc finger motifs that are critical for neurodevelopment [73, 77]. Evidence found that *ZSCAN31* might underlie several neurodevelopmental disorders, including ADHD, ASD, Tourette syndrome, and SCZ [82, 83]. *FBN3* encodes a member of the fibrillin protein family. Previous GWAS analysis have indicated associations between FBN3 and ADHD, brain morphology [74, 106].

Furthermore, our PRS analyses revealed ADHD PRS significantly predicted DD scores, providing additional evidence for a shared genetic basis between ADHD and short-term VSWM. Relationships between PRS and other three indexes failed to pass the permutation test, which might suggest the validity of DD, but not other indexes, as a neuropsychological endophenotype for ADHD. And this result was not surprising, because the delayed recall was one of the most consistent impaired functions reported in ADHD during RCFT task, and DD was supposed to demand the most WM load, able to reflect both encoding, maintenance, retrieval, organization, local and global processing [10, 14–16]. Our findings showed consistency with previous investigations found genetic correlations between ADHD and WM function [29, 107].

(3) Shared genetic basis between different part of VSWM.

Notably, one potentially shared genetic locus between DD, DI, and SI is rs56111147 located within the TCL1B gene, which encodes a T-Cell Leukemia/Lymphoma-1 family protein. In addition, the Hi-C data suggested that a chromatin loop can form between the genomic region (chr14:96,016,337–96026337, hg19) where rs56111147 is located and the promoter of *TCL1A* and *TCL6* (chr14:96,186,337–96,196,337, hg19). Although evidence mainly focused on the involvement of *TCLs* in hematological diseases and cancers, a previous large-scale GWAS (n = 542,934) study identified associations between myopia and *TCLs* [68]. Further researches need to be done to fully understand the role of *TCLs* play in VSWM.

Genes *FBN3* and *KLF7* were also shared in structure scores, i.e., SD and SI. *FBN3* encodes a member of the fibrillin protein family, mainly localized to extracellular microfibrils of developing skeletal elements, and responsible to Weill–Marchesani Syndrome and Marfan syndrome. Evidence also suggested its expression in the brain (UniProt/SwissProt) [108]. Although previous studies have not yet clarified the function *FBN3* plays in nervous system, GWAS studies identified associations between *FBN3* and ADHD, brain morphology, which implied its role in NDDs by altering brain development. *KLF7* encodes kruppel-like transcriptional regulator family, which regulates cell proliferation, differentiation, survival and contains three C2H2 zinc fingers at the C-terminus that mediate binding to GC-rich sites. *KLF7* plays a critical role in neuronal morphogenesis and survival of sensory neurons, and represses the corneal epithelium differentiation [76]. *KLF7* are also found to be associated with several psychiatric disorders, including ASD [109, 110], MDD [111] and sleep problems [112, 113]. A previous study reported that knockdowning *KLF7* in human brain organoids caused dysregulation of 517 ASD risk genes. Moreover, an increase of *KLF7* in the neural system in *KLF7* ± adult mice significantly rescued ASD symptoms and the expression of majority of dysregulated ASD genes [110]. According to previous literatures, *KLF7* might provide protective effects to the brain through regulating the neural axon plasticity and activation of the JAK2/STAT3 signaling pathway [114, 115]. While there is no research on the potential role KLF7 plays in VSWM or ADHD, our results encourage future studies.

(4) Forebrain commitment and dopamine transport are involved with VSWM.

The current study identified that forebrain neuron fate commitment and dopamine transport were significantly associated with DD and SD, respectively. Forebrain neuron fate commitment refers to the process in which the developmental fate of a cell in frontal lobe becomes restricted, which mostly occurs prenatally [116, 117]. Studies have demonstrated that disturbances in neural differentiation have profound effects on brain development and function, which might contribute to psychiatric disorders and cognitive deficits in later life through a variety of mechanisms, including aberrant neural migration, impaired synaptogenesis, and disruptions in neurotransmitter systems, et, al. Of particular interest are the neurotransmitter systems, where disruptions in the expression of genes in this pathway, including *PAX6, BCL11B, GATA2, ASCL1, TBL1, NKX2.1* could result in abnormal GABAergic, dopaminergic, and glutamatergic neuron development [118–124], and are associated with cognitive deficits [122, 125–128]. A possible underling pathophysiological pathway is that the relevant genes might affect the excitatory/inhibitory balance [122–124], which is also considered as mechanisms in most psychiatry disorders including ADHD [129–131]

To conclude, our results indicated an early onset of altered neural substrates occurred during neuronal differentiation underlying VSWM and further emphasized the involvement of dopaminergic circuit.

Our study should be interpreted in light of some limitations. Firstly, we solely utilized the RCFT to evaluate VSWM. As previous studies have demonstrated, the RCFT is more sensitive to active WM processes [132]. Besides, EF is intensively required in RCFT task, which might be a confounding factor [12]. Therefore, future studies are encouraged to incorporate multiple VSWM tests to provide a more comprehensive assessment. Secondly, although we have performed interaction analyses to explore potential confounding effects caused by age, it remains difficult to fully address these effects. Future studies should take this issue into consideration through subgroup analyses or other methods with a larger sample size to provide more comprehensive insights.

It is worth noting that, as highlighted by previous findings, complex traits are often influenced by thousands or more genetic variants that exert their effects cumulatively. The "nominal" variants are believed to contribute a considerable proportion of the overall heritability [28, 133]. Thus, although no significant variants were detected by the current analysis, the suggestive variants might still provide further insights and lay a foundation for future studies in this area.

## Conclusion

This study represents a genome-wide association study of VSWM, in which several genes, including *TCL1B, KLF7,* and *FBN3,* were identified as potentially related to VSWM. In addition, the brain-expressed gene *RAB11FIP1* was found to be significantly associated with DD. The results of polygenic risk score analyses provided evidence of shared genetic underpinnings between ADHD and VSWM, shedding light on the genetic basis of these conditions and potentially informing clinical practice in the future.

## Data Availability

Not applicable.

## References

[1] Thomas R, Sanders S, Doust J, Beller E, Glasziou P (2015). Prevalence of attention-deficit/hyperactivity disorder: a systematic review and meta-analysis. Pediatrics.

[2] Faraone SV (2015). Attention-deficit/hyperactivity disorder. Nat Rev Dis Primers.

[3] Barkley RA (2006). Attention-deficit hyperactivity disorder.

[4] Kasper LJ, Alderson RM, Hudec KL (2012). Moderators of working memory deficits in children with attention-deficit/hyperactivity disorder (ADHD): a meta-analytic review. Clin Psychol Rev.

[5] Willcutt EG, Doyle AE, Nigg JT, Faraone SV, Pennington BF (2005). Validity of the executive function theory of attention-deficit/hyperactivity disorder: a meta-analytic review. Biol Psychiatry.

[6] Martinussen R, Hayden J, Hogg-Johnson S, Tannock R (2005). A meta-analysis of working memory impairments in children with attention-deficit/hyperactivity disorder. J Am Acad Child Adolesc Psychiatry.

[7] Alderson RM, Kasper LJ, Hudec KL, Patros CHG (2013). Attention-deficit/hyperactivity disorder (ADHD) and working memory in adults: a meta-analytic review. Neuropsychology.

[8] Patros CHG, Alderson RM, Hudec KL, Tarle SJ, Lea SE (2017). Hyperactivity in boys with attention-deficit/hyperactivity disorder: The influence of underlying visuospatial working memory and self-control processes. J Exp Child Psychol.

[9] Dovis S, Van der Oord S, Huizenga HM, Wiers RW, Prins PJM (2015). Prevalence and diagnostic validity of motivational impairments and deficits in visuospatial short-term memory and working memory in ADHD subtypes. Eur Child Adolesc Psychiatry.

[10] Hyun GJ (2018). Visuospatial working memory assessment using a digital tablet in adolescents with attention deficit hyperactivity disorder. Comput Methods Programs Biomed.

[11] Rey A (1941). L’examen psychologique dans les cas d’encephalopathie traumatique. Arch Psychol.

[12] Shin M-S, Park S-Y, Park S-R, Seol S-H, Kwon JS (2006). Clinical and empirical applications of the rey-osterrieth complex figure test. Nat Protoc.

[13] Zhang X, Lv L, Min G, Wang Q, Zhao Y, Li Y (2021). Overview of the complex figure test and its clinical application in neuropsychiatric disorders, including copying and recall. Front Neurol.

[14] Shuai L, Chan RCK, Wang Y (2011). Executive function profile of Chinese boys with attention-deficit hyperactivity disorder: different subtypes and comorbidity. Arch Clin Neuropsychol.

[15] Shin M-S, Kim Y-H, Cho S-C, Kim B-N (2003). Neuropsychologic characteristics of children with attention-deficit hyperactivity disorder (ADHD), learning disorder, and tic disorder on the Rey-Osterreith Complex Figure. J Child Neurol.

[16] Seidman LJ (1995). Performance of children with ADHD on the Rey-Osterrieth complex figure: a pilot neuropsychological study. J Child Psychol Psychiatry.

[17] Lemvigh CK (2022). Heritability of specific cognitive functions and associations with schizophrenia spectrum disorders using CANTAB: a nation-wide twin study. Psychol Med.

[18] Zhou H (2018). Heritability estimates of spatial working memory and set-shifting in a healthy Chinese twin sample: A preliminary study: Heritability of spatial working memory. Psych J.

[19] Blokland GAM (2008). Quantifying the heritability of task-related brain activation and performance during the N-back working memory task: a twin fMRI study. Biol Psychol.

[20] Shang C-Y, Gau SS-F (2014). Association between the DAT1 gene and spatial working memory in attention deficit hyperactivity disorder. Int J Neuropsychopharm.

[21] Thissen AJAM (2015). The role of age in association analyses of ADHD and related neurocognitive functioning: A proof of concept for dopaminergic and serotonergic genes. Am J Med Genet Pt B.

[22] Zilles D (2012). Genetic polymorphisms of 5-HTT and DAT but not COMT differentially affect verbal and visuospatial working memory functioning. Eur Arch Psychiatry Clin Neurosci.

[23] Leo D (2018). Pronounced hyperactivity, cognitive dysfunctions, and bdnf dysregulation in dopamine transporter knock-out rats. J Neurosci.

[24] Trampush JW, Jacobs MM, Hurd YL, Newcorn JH, Halperin JM (2014). Moderator effects of working memory on the stability of ADHD symptoms by dopamine receptor gene polymorphisms during development. Dev Sci.

[25] Dumontheil I, Kilford EJ, Blakemore S-J (2020). Development of dopaminergic genetic associations with visuospatial, verbal and social working memory. Dev Sci.

[26] Dumontheil I (2011). Influence of the COMT genotype on working memory and brain activity changes during development. Biol Psychiatry.

[27] Dumontheil I, Jensen SKG, Wood NW, Meyer ML, Lieberman MD, Blakemore S-J (2014). Preliminary investigation of the influence of dopamine regulating genes on social working memory. Soc Neurosci.

[28] Boyle EA, Li YI, Pritchard JK (2017). An expanded view of complex traits: from polygenic to omnigenic. Cell.

[29] Donati G, Dumontheil I, Meaburn EL (2019). Genome-wide association study of latent cognitive measures in adolescence: genetic overlap with intelligence and education. Mind Brain Educ.

[30] Soo CC (2023). Genome-wide association study of population-standardised cognitive performance phenotypes in a rural South African community. Commun Biol.

[31] Blokland GAM (2017). Genome-wide association study of working memory brain activation. Int J Psychophysiol.

[32] Lahti J (2022). Genome-wide meta-analyses reveal novel loci for verbal short-term memory and learning. Mol Psychiatry.

[33] Kamiński J, Sullivan S, Chung JM, Ross IB, Mamelak AN, Rutishauser U (2017). Persistently active neurons in human medial frontal and medial temporal lobe support working memory. Nat Neurosci.

[34] Constantinidis C, Wang X-J (2004). A neural circuit basis for spatial working memory. Neuroscientist.

[35] Rezayat E, Dehaqani M-RA, Clark K, Bahmani Z, Moore T, Noudoost B (2021). Frontotemporal coordination predicts working memory performance and its local neural signatures. Nat Commun.

[36] Roussy M (2022). Stable working memory and perceptual representations in macaque lateral prefrontal cortex during naturalistic vision. J Neurosci.

[37] Cropley VL, Fujita M, Innis RB, Nathan PJ (2006). Molecular imaging of the dopaminergic system and its association with human cognitive function. Biol Psychiat.

[38] Levy R, Goldman-Rakic PS (2000). Segregation of working memory functions within the dorsolateral prefrontal cortex. Exp Brain Res.

[39] Jung M (2019). The effects of COMT polymorphism on cortical thickness and surface area abnormalities in children with ADHD. Cereb Cortex.

[40] Vijayraghavan S, Wang M, Birnbaum SG, Williams GV, Arnsten AFT (2007). Inverted-U dopamine D1 receptor actions on prefrontal neurons engaged in working memory. Nat Neurosci.

[41] McNab F (2009). Changes in cortical dopamine D1 receptor binding associated with cognitive training. Science.

[42] Mehta MA, Owen AM, Sahakian BJ, Mavaddat N, Pickard JD, Robbins TW (2000). Methylphenidate enhances working memory by modulating discrete frontal and parietal lobe regions in the human brain. J Neurosci.

[43] Yang L, Wang Y-F, Qian Q-J, Biederman J, Faraone SV (2004). DSM-IV Subtypes of adhd in a chinese outpatient samplE. J Am Acad Child Adolesc Psychiatry.

[44] Yang L (2013). Polygenic transmission and complex neuro developmental network for attention deficit hyperactivity disorder: Genome-wide association study of both common and rare variants. Am J Med Genet.

[45] Wechsler D (2003). Wechsler Intelligence Scale for Children.

[46] Chen H, Keith TZ, Weiss L, Zhu J, Li Y (2010). Testing for multigroup invariance of second-order WISC-IV structure across China, Hong Kong, Macau, and Taiwan. Personality Individ Differ.

[47] Wigginton JE, Cutler DJ, Abecasis GR (2005). A note on exact tests of hardy-weinberg equilibrium. Am J Hum Genet.

[48] Liu EY, Li M, Wang W, Li Y (2013). MaCH-admix: genotype imputation for admixed populations. Genet Epidemiol.

[49] Price AL, Patterson NJ, Plenge RM, Weinblatt ME, Shadick NA, Reich D (2006). Principal components analysis corrects for stratification in genome-wide association studies. Nat Genet.

[50] Chang CC, Chow CC, Tellier LC, Vattikuti S, Purcell SM, Lee JJ (2015). Second-generation PLINK: rising to the challenge of larger and richer datasets. GigaSci.

[51] Shaun Purcell and Christopher Chang. “PLINK.” Available: www.cog-genomics.org/plink/1.9/

[52] Purcell S (2007). PLINK: a tool set for whole-genome association and population-based linkage analyses. The American J Hum Genet.

[53] “VannoPortal: multiscale functional annotation of human genetic variants for interrogating molecular mechanism of traits and diseases. Nucleic Acids Research. Oxford Academic.” https://academic.oup.com/nar/article/50/D1/D1408/6376021 Accessed. 27 Feb 2023. 10.1093/nar/gkab853 PMC8728305 34570217

[54] Quan C, Ping J, Lu H, Zhou G, Lu Y (2022). 3DSNP 2.0: update and expansion of the noncoding genomic variant annotation database. Nucleic Acids Res.

[55] GeneCards, “The human gene database.” https://www.genecards.org/. Accessed. 27 Feb 2023.

[56] Stelzer G (2016). The genecards suite: from gene data mining to disease genome sequence analyses. Curr Protoc Bioinformatics.

[57] de Leeuw CA, Mooij JM, Heskes T, Posthuma D (2015). MAGMA: generalized gene-set analysis of GWAS data. PLoS Comput Biol.

[58] Warde-Farley D (2010). The GeneMANIA prediction server: biological network integration for gene prioritization and predicting gene function. Nucleic Acids Res.

[59] Shannon P (2003). Cytoscape: a software environment for integrated models of biomolecular interaction networks. Genome Res.

[60] ADHD Working Group of the Psychiatric Genomics Consortium (PGC) (2019). Discovery of the first genome-wide significant risk loci for attention deficit/hyperactivity disorder. Nat Genet.

[61] Stahl EA (2019). Genome-wide association study identifies 30 loci associated with bipolar disorder. Nat Genet.

[62] Sanchez-Roige S (2019). Genome-wide association study meta-analysis of the alcohol use disorders identification test (AUDIT) in two population-based cohorts. Am J Psychiatry.

[63] Autism Spectrum Disorder Working Group of the Psychiatric Genomics Consortium (2019). Identification of common genetic risk variants for autism spectrum disorder. Nat Genet.

[64] Mapping genomic loci implicates genes and synaptic biology in schizophrenia - PubMed.2023 https://pubmed.ncbi.nlm.nih.gov/35396580/ Accessed. 21 Jan 2023. 10.1038/s41586-022-04434-5 PMC9392466 35396580

[65] International Obsessive Compulsive Disorder Foundation Genetics Collaborative (IOCDF-GC) and OCD Collaborative Genetics Association Studies (OCGAS) (2018). Revealing the complex genetic architecture of obsessive-compulsive disorder using meta-analysis. Mol Psychiatry.

[66] Giannakopoulou O (2021). The genetic architecture of depression in individuals of east asian ancestry: a genome-wide association study. JAMA Psychiat.

[67] Choi SW, P. F. OReilly.  (2019). PRSice-2: polygenic risk score software for biobank-scale data. GigaScience.

[68] Hysi PG (2020). Meta-analysis of 542,934 subjects of European ancestry identifies new genes and mechanisms predisposing to refractive error and myopia. Nat Genet.

[69] Re A, De Franchis V, Cornoldi C (2010). Working memory control deficit in kindergarten ADHD children. Child Neuropsychol.

[70] Roussy M, Mendoza-Halliday D, Martinez-Trujillo JC (2021). Neural Substrates of Visual Perception and Working Memory: Two Sides of the Same Coin or Two Different Coins?. Front Neural Circuits..

[71] Fuster JM, Alexander GE (1971). Neuron activity related to short-term memory. Science.

[72] Mendoza-Halliday D, Martinez-Trujillo JC (2017). Neuronal population coding of perceived and memorized visual features in the lateral prefrontal cortex. Nat Commun.

[73] Al-Naama N, Mackeh R, Kino T (2020). C2H2-Type zinc finger proteins in brain development, neurodevelopmental, and other neuropsychiatric disorders: systematic literature-based analysis. Front Neurol.

[74] Hawi Z (2018). A case-control genome-wide association study of ADHD discovers a novel association with the tenascin R (TNR) gene. Transl Psychiatry.

[75] Ben-Gigi L (2015). Astrogliosis induced by brain injury is regulated by Sema4B phosphorylation. Eneuro.

[76] Klein RH (2017). Characterization of enhancers and the role of the transcription factor KLF7 in regulating corneal epithelial differentiation. J Biol Chem.

[77] Liu Q, Shi Z, Liu X, Xiao H (2020). Correlation between the coexpression of zinc finger and SCAN domain-containing protein 31 and transcriptional activator with PDZ-binding motif and prognosis in hepatocellular carcinoma. Ann Transl Med.

[78] Jia X (2022). De novo variants in genes regulating stress granule assembly associate with neurodevelopmental disorders. Sci Adv.

[79] Demontis D (2019). Discovery of the first genome-wide significant risk loci for attention deficit/hyperactivity disorder. Nat Genet.

[80] Aebi M (2016). Gene-set and multivariate genome-wide association analysis of oppositional defiant behavior subtypes in attention-deficit/hyperactivity disorder. Am J Med Genet.

[81] Rao S, Baranova A, Yao Y, Wang J, Zhang F (2022). Genetic relationships between attention-deficit/hyperactivity disorder, autism spectrum disorder, and intelligence. Neuropsychobiology.

[82] Goes FS (2015). Genome-wide association study of schizophrenia in Ashkenazi Jews. Am J Med Genet.

[83] Lee PH (2019). Genomic relationships, novel loci, and pleiotropic mechanisms across eight psychiatric disorders. Cell.

[84] Trubetskoy V (2022). Mapping genomic loci implicates genes and synaptic biology in schizophrenia. Nature.

[85] Karlsson Linnér R (2021). Multivariate analysis of 1.5 million people identifies genetic associations with traits related to self-regulation and addiction. Nat Neurosci.

[86] Han X (2020). Association of myopia and intraocular pressure with retinal detachment in european descent participants of the uk biobank cohort: a mendelian randomization study. JAMA Ophthalmol.

[87] Gao XR, Huang H, Nannini DR, Fan F, Kim H (2018). Genome-wide association analyses identify new loci influencing intraocular pressure. Hum Mol Genet.

[88] Mandell JT, de Rivero Vaccari JP, Sabater AL, Galor A (2023). The inflammasome pathway: a key player in ocular surface and anterior segment diseases. Surv Ophthalmol.

[89] The Consortium for Refractive Error and Myopia (2020). Meta-analysis of 542,934 subjects of European ancestry identifies new genes and mechanisms predisposing to refractive error and myopia. Nat Genet.

[90] Xue Z (2022). Genome-wide association meta-analysis of 88,250 individuals highlights pleiotropic mechanisms of five ocular diseases in UK Biobank. EBioMedicine.

[91] Pickrell JK, Berisa T, Liu JZ, Ségurel L, Tung JY, Hinds DA (2016). Detection and interpretation of shared genetic influences on 42 human traits. Nat Genet.

[92] Tideman JWL (2021). Evaluation of shared genetic susceptibility to high and low myopia and hyperopia. JAMA Ophthalmol.

[93] The CREAM Consortium (2018). Genome-wide association meta-analysis highlights light-induced signaling as a driver for refractive error. Nat Genet.

[94] Hendee K, Wang LW, Reis LM, Rice GM, Apte SS, Semina EV (2017). Identification and functional analysis of an *ADAMTSL1* variant associated with a complex phenotype including congenital glaucoma, craniofacial, and other systemic features in a three-generation human pedigree. Hum Mutat.

[95] Ramshekar A, Hartnett ME (2021). Vascular Endothelial Growth Factor Signaling in Models of Oxygen-Induced Retinopathy: Insights Into Mechanisms of Pathology in Retinopathy of Prematurity. Front Pediatr.

[96] Suarez MF (2021). Transcriptome analysis of pterygium and pinguecula reveals evidence of genomic instability associated with chronic inflammation. IJMS.

[97] Li AS (2021). Whole-exome sequencing of patients with posterior segment uveitis. Am J Ophthalmol.

[98] Jiang X (2020). Fine-mapping and cell-specific enrichment at corneal resistance factor loci prioritize candidate causal regulatory variants. Commun Biol.

[99] Siri SO (1867). 2020 Decrease of Rab11 prevents the correct dendritic arborization, synaptic plasticity and spatial memory formation. Biochim Biophys Acta Mol Cell Res.

[100] Krämer R, Rode S, Rumpf S (2019). Rab11 is required for neurite pruning and developmental membrane protein degradation in Drosophila sensory neurons. Dev Biol.

[101] Clark BS, Miesfeld JB, Flinn MA, Collery RF, Link BA (2021). Dynamic polarization of rab11a modulates crb2a localization and impacts signaling to regulate retinal neurogenesis. Front Cell Dev Biol.

[102] Murciano A, Zamora J, Lopezsanchez J, Frade J (2002). Interkinetic nuclear movement may provide spatial clues to the regulation of neurogenesis. Mol Cell Neurosci.

[103] Xiong B (2012). Crag is a GEF for Rab11 required for rhodopsin trafficking and maintenance of adult photoreceptor cells. PLoS Biol.

[104] Satoh AK, O’Tousa JE, Ozaki K, Ready DF (2005). Rab11 mediates post-Golgi trafficking of rhodopsin to the photosensitive apical membrane of Drosophila photoreceptors. Development.

[105] Neale BM (2010). Meta-analysis of genome-wide association studies of attention-deficit/hyperactivity disorder. J Am Acad Child Adolesc Psychiatry.

[106] van der Meer D (2021). The genetic architecture of human cortical folding. Sci Adv.

[107] Martin J, Hamshere ML, Stergiakouli E, O’Donovan MC, Thapar A (2015). Neurocognitive abilities in the general population and composite genetic risk scores for attention-deficit hyperactivity disorder. J Child Psychol Psychiatry.

[108] The UniProt Consortium (2023). UniProt: the universal protein knowledgebase in 2023. Nucleic Acids Res.

[109] Tian H (2022). Krüppel-like factor 7 deficiency causes autistic-like behavior in mice via regulating Clock gene. Cell Biosci.

[110] Tian H (2022). Krüppel-like transcription factor 7 is a causal gene in autism development. Int J Mol Sci.

[111] Yao X (2021). Integrative analysis of genome-wide association studies identifies novel loci associated with neuropsychiatric disorders. Transl Psychiatry.

[112] Jansen PR (2019). Genome-wide analysis of insomnia in 1,331,010 individuals identifies new risk loci and functional pathways. Nat Genet.

[113] Watanabe K (2022). Genome-wide meta-analysis of insomnia prioritizes genes associated with metabolic and psychiatric pathways. Nat Genet.

[114] Li W-Y (2017). AAV-KLF7 promotes descending propriospinal neuron axonal plasticity after spinal cord injury. Neural Plast.

[115] Li W-Y (2022). Krüppel-like factor 7 attenuates hippocampal neuronal injury after traumatic brain injury. Neural Regen Res.

[116] Chhetri PK, Das JM (2022). Neuroanatomy, neural tube development and stages.

[117] Temple S (2001). The development of neural stem cells. Nature.

[118] Konishi T (2013). Benign hereditary chorea: dopaminergic brain imaging in patients with a novel intronic NKX2.1 gene mutation. J Neurol.

[119] Wullimann MF, Rink E (2001). Detailed immunohistology of Pax6 protein and tyrosine hydroxylase in the early zebrafish brain suggests role of Pax6 gene in development of dopaminergic diencephalic neurons. Brain Res Dev Brain Res.

[120] Gustorff C, Scheuer T, Schmitz T, Bührer C, Endesfelder S (2021). GABAB receptor-mediated impairment of intermediate progenitor maturation during postnatal hippocampal neurogenesis of newborn rats. Front Cell Neurosci.

[121] Achim K, Salminen M, Partanen J (2014). Mechanisms regulating GABAergic neuron development. Cell Mol Life Sci.

[122] Dulcis D, Lippi G, Stark CJ, Do LH, Berg DK, Spitzer NC (2017). Neurotransmitter switching regulated by mirnas controls changes in social preference. Neuron.

[123] Du T, Xu Q, Ocbina PJ, Anderson SA (2008). NKX2.1 specifies cortical interneuron fate by activating Lhx6. Development.

[124] Georgala PA, Carr CB, Price DJ (2011). The role of Pax6 in forebrain development. Dev Neurobiol.

[125] Haji N, Riebe I, Aguilar-Valles A, Artinian J, Laplante I, Lacaille J-C (2020). Tsc1 haploinsufficiency in Nkx21 cells upregulates hippocampal interneuron mTORC1 activity, impairs pyramidal cell synaptic inhibition, and alters contextual fear discrimination and spatial working memory in mice. Molecular Autism..

[126] Graziola F, Garone G, Grasso M, Schirinzi T, Capuano A (2021). Working memory, attention and planning abilities in NKX2.1-related chorea. Parkinsonism Relat Disord.

[127] Jung S (2020). Autophagic death of neural stem cells mediates chronic stress-induced decline of adult hippocampal neurogenesis and cognitive deficits. Autophagy.

[128] Thompson PJ (2004). Cognitive functioning in humans with mutations of the PAX6 gene. Neurology.

[129] Satterstrom FK (2020). Large-scale exome sequencing study implicates both developmental and functional changes in the neurobiology of autism. Cell.

[130] Pocklington AJ (2015). Novel findings from cnvs implicate inhibitory and excitatory signaling complexes in schizophrenia. Neuron.

[131] Morello F (2020). ADHD-like behaviors caused by inactivation of a transcription factor controlling the balance of inhibitory and excitatory neuron development in the mouse anterior brainstem. Transl Psychiatry.

[132] Fastame MC (2020). Visual and spatial working memory skills implicated in copying and drawing from memory of the Rey-Osterrieth Complex Figure: What relationship in school-aged children?. Cogn Dev.

[133] Yang J (2010). Common SNPs explain a large proportion of the heritability for human height. Nat Genet.

